# Messenger-based chat counseling for young people in times of personal and global crises: evaluating the long-term feasibility of “krisenchat”

**DOI:** 10.3389/fdgth.2026.1787638

**Published:** 2026-08-03

**Authors:** Juliane Hug, Elisabeth Kohls, Sabrina Baldofski, Jelena Scheider, Shadi Saee, Melanie Eckert, Richard Wundrack, Christine Rummel-Kluge

**Affiliations:** 1 Department of Psychiatry and Psychotherapy, Medical Faculty, Leipzig University, Leipzig, Germany; 2 Department of Psychiatry and Psychotherapy, University Leipzig Medical Center, Leipzig University, Leipzig, Germany; 3 krisenchat gGmbH, Berlin, Germany

**Keywords:** adolescents, chat counseling, e-Mental-Health, helpline, online intervention

## Abstract

**Background:**

Adolescence is a critical period for the onset of mental health problems, a situation further exacerbated by recent global crises. This underscores the importance of low-threshold, anonymous online counseling services. *Krisenchat*, founded in Germany in 2020 following the COVID-19 outbreak, provides such support. While prior research has offered cross-sectional insights, little is known about changes in user characteristics and measures of the service's feasibility over time, especially regarding the chat topics addressed and in recognition of all gender identities.

**Objective:**

This study examined the long-term feasibility and scalability of *krisenchat* over three years (2020–2023) and explored changes over time in user characteristics, service feasibility and satisfaction, emotional distress, and counseling topics.

**Methods:**

This cross-sectional study analyzed retrospective, naturalistic, anonymous data on sociodemographic variables, utilization behavior, and user satisfaction from *krisenchat* users over three selected, consecutive time periods (Oct 2020–Mar 2021, *N* = 4,029; Oct 2021–Mar 2022, *N* = 6,647; Oct 2022–Mar 2023, *N* = 5,218). *χ*^2^ tests were used to identify associations between users’ sociodemographic characteristics and chat topics over time. Kruskal–Wallis H tests examined measures such as age, emotional distress, user satisfaction, and perceived helpfulness. This was followed by an exploratory analysis of chat topics across time and genders.

**Results:**

Users reported significantly reduced emotional distress after chat sessions. Across all periods, the average user was 17 years old and female, while engagement among males and gender-diverse individuals increased over time. *krisenchat* remained feasible and well-received in all time points. Key chat topics included psychosocial distress, mental health symptoms and emotional distress. Chat topics evolved, towards more psychosocial concerns.

**Conclusion:**

This study highlights the robustness of krisenchat from 2020 to 2023, underscoring its sustained relevance in both pandemic and post-pandemic contexts. By integrating gender analyses and exploring chat topics over time, the study reveals novel insights into shifting user needs, such as increased psychosocial distress and relational concerns in post-pandemic periods. Observed trends, such as increased engagement among males and diverse users, emphasize the importance of tailored outreach efforts. These findings enrich the evidence base for digital mental health services and offer insights for future interventions.

**Study Registration:**

DRKS00026671

## Introduction

The majority of mental disorders develop and manifest before the age of 24 (1, 2), marking adolescence as a phase particularly vulnerable to external stressors. Since 2020, the world has been impacted by a series of significant stressors, so-called collective life events (3): three years of a pandemic, a major war in Europe, an ensuing economic crisis, and the visible effects of climate change all of which have profoundly altered young people's lives. This phenomenon has been referred to as a polycrisis: “the presence of multiple near-simultaneous shocks, with strong interdependencies among them” (4). COVID-19-related physical distancing measures and other non-pharmaceutical interventions (NPIs; e.g., school closings, stay-at-home orders, and restrictions on physical gatherings) led to abrupt and significant changes in young people's daily lives and social relationships. These changes led to an increase in mental health challenges, a worsening of pre-existing conditions, disruptions in continuity of care, and an exacerbation of the treatment gap among young individuals (5, 6). Low-threshold online support services for children and young adults, such as chat counseling, have been increasingly developed and utilized since the onset of the COVID-19 pandemic (7, 8), with a notable rise in uptake from 2020 onward (9). Moreover, younger individuals are disproportionately experiencing climate distress and bearing the mental health burdens associated with climate change (10). In response to these challenges, the polycrisis has increased the demand for low-threshold, easily accessible mental health support, particularly for vulnerable groups such as young men and LGBTQIA+ individuals. These populations often exhibit elevated psychological distress and low help-seeking behavior (11). Diverse and cisgender users face distinct challenges in approaching online counseling services (12).

Strengthening the evidence base for online counseling programs in the context of personal crisis support in times of global crises and its impact on young people's mental health is essential. *k*risenchat is one such online intervention launched in Germany in May 2020, shortly after the first pandemic lockdown. It is a messenger-based psychological counseling service for children, adolescents, and young adults from 8 to 24 years. Previous research demonstrated the feasibility and acceptability of krisenchat in a large naturalistic sample of 6,962 users (13). The average user was 17 years old and female, with the most common chat topics being psychiatric symptoms (60.1%), psychosocial concerns (34.0%), and emotional distress (30.2%) (13). The service has shown potential in providing rapid stress relief in acute crises such as suicidality (14). Additionally, almost half of users followed recommendations for seeking help and over 72% stated that they had an appointment or talk with the service or person recommended (15). Despite being at increased risk of suicidality and being less likely to seek traditional mental health support, young men showed high acceptance of the 24/7 online counseling service *krisenchat*, while exhibiting partly gender-specific patterns of service use and presenting concerns (16). Since the male youth is more reluctant to reach out and seek help, *krisenchat* has a dedicated a psychologist for “Jungenarbeit” [work with boys]. Between 2023 and 2026, a German Health Insurance has funded additional dedicated efforts of providing general psychosocial education targeting boys and young men via social media platforms like Reddit, Twitch, Discord, and TikTok. While the primary goal of this project was to reach out to the male youth more successfully, it was not to get more male youth into *krisenchat's* one-on-one crisis counselling service.

Despite these findings, prior analyses of krisenchat have been limited to cross-sectional data at single points in time. Limited research exists on the long-term use of crisis services, particularly during global crises. This is especially true for marginalized groups (e.g., diverse gender identities) and online text-based counseling approaches (17).

This present study therefore aims to (1) analyze user characteristics, user satisfaction, and impact on emotional distress of the *krisenchat* service in a current, post-pandemic naturalistic user sample; (2) examine the development of user characteristics and satisfaction over time; and (3) explore changes in chat topics, particularly pandemic- and crisis-related themes, with respect to all gender identities. We hypothesized that user satisfaction will remain consistently high, while crisis-related topics will shift over time due to ongoing polycrisis and associated mental health problems.

Given the restricted age range of *krisencha*t users (8–24 years), representing a developmental period characterized by heightened vulnerability to mental health problems, subgroup analyses focused on gender, which was a primary objective of this study due to well-established differences in mental health burden and help-seeking behavior.

## Material and methods

### Description of krisenchat

*krisenchat (*https://www.krisenchat.de*)* is a non-profit anonymous chat service designed to assist children and young adults in acute crises. It is available 24/7 at no cost via WhatsApp or text messaging. Counselors are volunteers and employees with backgrounds in psychology, psychotherapy or social work. *krisenchat* was launched in May 2020 and since has seen a growth in its number of users. *krisenchat*'s crisis counselling service is maintained and operated by a mix of volunteer and staff psychologists, psychotherapists, and social workers balancing capacities to the regular weekday and time of day demand fluctuations and a baseline around the clock availability. The counselling chat platform is continuously improved and maintained by a team of internal developers. For a more detailed description of the service, please refer to (13).

Since its launch, *krisenchat* recorded a growth in overall usage: The number of active counselors has steadily increased, from 430 in 2021 to 637 in 2022, and further to 843 in 2023. The number of users receiving consultations grew from 12,957 in 2021 to 17,397 in 2022 but remained nearly the same in 2023 at 17,456. The total number of consultations increased from 33,600 in 2021 to 39,157 in 2022 but slightly declined to 37,705 in 2023. Counselor data were obtained from the company's operational databases for the years 2021, 2022, and 2023.

During the observation period, *krisenchat* underwent continuous organizational development. Alongside the expansion of the counselling service, ongoing outreach and marketing activities were implemented to increase awareness and accessibility among young people. In parallel, the volunteer training concept and quality assurance procedures were continuously refined through the introduction of standardized counselling guidelines, regular supervision and feedback processes, and expanded support structures for counsellors.

### Participants and procedure

For the current analysis, data were extracted for three time periods to (1) control for seasonal effects while they also (2) represent specific phases of the polycrisis, mainly phases of the pandemic:

**1st October 2020–31st March 2021** (TP1, *lockdown phase*): The period was marked by the second COVID-19 wave in Germany, which peaked towards the end of 2020. With 2,158,013 reported COVID-19 cases, the second wave was more severe than the first. *krisenchat* was launched in May 2020, making this the first year of service.
**1st October 2021–31st March 2022** (TP2, *pandemic phase*): A total of 120 different measures were implemented in Germany to combat the COVID-19 pandemic, including a vaccination campaign, mask mandates, testing infrastructure, quarantine requirements, social distancing, and travel restrictions. These national measures officially ended by April 2022. February 2022 was marked by the beginning of the war against Ukraine, leading to a massive influx of refugees into Germany.
**1st October 2022–31st Mach 2023** (TP3, *post pandemic phase*): By January 2023, scientists in Germany had largely defined COVID-19 as a non-lethal respiratory pathogen. In May 2023, the WHO lifted the “international health emergency,” and by that time, most countries, including Germany, had returned to pre-pandemic life.

The final sample consisted of *N* = 4,029 for TP1, *N* = 6,647 for TP2 and *N* = 5,218 for TP3.

The data of all three time points included (1) automatically-collected **metadata** of each chat (e.g., date and duration of the session in form of time stamps) and (2) **information documented by the counsellors** (i.e., age, gender, chat topic) and (3) deliberate **user feedback** obtained through an automatically sent survey invitation after the first chat session. Users received the survey invitation (with a link to the survey) via chat 6 h after counseling if the session included at least 30 messages and was not considered “at-risk” by the counselling team (due to suspected self-harm, suicidality and/or child welfare endangerment). If users attended multiple sessions, they only received the survey once after the first session. For this study, data from the German chat platform were extracted and analyzed. Informed consent was obtained via an opt-in function before survey participation. The Ethics Committee of the Medical Faculty, University of Leipzig, granted ethical approval on August 3, 2021 (file reference: 372/21-ek).

### Measures

#### User characteristics and chat topics (by counsellors)

Chat counselors collected information provided by users as well as details about them (i.e., age, gender, concerns identified during counseling, current use of professional treatment). Users' concerns or problems were categorized by chat topics that were originally aggregated into eight umbrella categories {psychiatric symptoms, psychosocial distress, emotional distress, sexual harassment, violence, education, LGBTQIA+, and COVID-19; see [Eckert et al. (13),] for further details}. If users returned within one time period, all topics were aggregated by the time of data extraction. In the first data period, 25 different topic tags were available, in the second and the third data period more differentiated tags as well as war-specific tags resulted in 60 and 61 tags, respectively. All topic tags were aggregated to keep the original overall 8 umbrella categories across all time periods [see Eckert et al. (13) and Table 1]. In the *krisenchat* database, some topic tags (see “Topics” in Table 1) were modified or added to the main categories (“Category” in Table 1) over time, while the 8 umbrella categories remained unchanged (see Table 1).

**Table 1:** Overview of chat categories and topics across the three time points.

	Category	Included topics Oct 20–Mar 21 (TP1)	Additional or modified topics Oct 21–Mar 22 (TP2)	Additional or modified topics Oct 22**–**Mar 23 (TP3)
1	Mental Health Symptoms	Self-harm, suicidal ideation/suicidality, addictive behavior, eating disorder symptoms, flashbacks, anxiety, obsessive-compulsive behavior	Borderline symptoms, autism symptoms, panic attacks, psychotic symptoms, psychosomatic/sleep disorder symptoms	
2	Psychosocial Distress	School-related problems, mobbing and bullying, family-related problems, mental health problems of others, performance pressure/high expectations	Work-related problems, friendship-related problems, problems related to partnership, fear of the future, education-related problems, physical health problems of others, self-harm of others, career-related performance pressure/high expectations, performance pressure/high expectations related to other topics, suffering related to online platforms/social media, stress	Toxic relationship, acutely impaired parent-child relationship, discrimination
3	Emotional Distress	Grief, lovesickness, anger, loneliness, selfworth, neglect^a^	Guilt	
4	Sexual Harassment	Sexual violence, sexual harassment		
5	Violence	Violence	Family, physical, partner, or psychological violence	
6	Covid	Covid/Lockdown		
7	Education	Psychoeducation	Coping with own diagnosis	
8	LGBTQIA+	LGBTQIA+		
9	War^b^			

a Topic was deleted in the second and third time period*.* More specific tags were available in the second and in the last time period (e.g., war) but were excluded from the current analyses.

b Topic was newly introduced at timepoint 3 (9th category).

The introduction and definition of potential chat topics is an annual reiterative process sensitive to the feedback of the counselors' needs and observations regarding crisis trends, e.g., in relation to the invasion of Ukraine, war and refugee related topics were added. Based on this feedback, topics are selected and revised by a group of *krisenchat* internal stakeholders bringing together service relevant, clinical, and academic expertise. Because *krisenchat* does not carry out a structured medical examination or make a diagnosis during counselling, chat topic definitions focus on reported indicators and common symptoms, e.g., instead of a topic tag “drug abuse” the tag reads “substance use”.

#### User satisfaction and emotional distress (by feedback questionnaire, self-report)

##### User satisfaction

The feedback questionnaire contained similar items across all three periods, though a few items changed after the first data collection (TP1) due to internal changes in quality management and data collection. User satisfaction was operationalized by (a) perceived helpfulness of the service and (b) the likelihood of recommending it to others. Perceived helpfulness was measured with one item asking whether *krisenchat* was able to help the user with their concern. Participants rated the helpfulness on a four-point Likert scale from 0 = “no”, 1 = “rather no”, 2 = “rather yes” to 3 = “yes” in the last two time periods (TP2, TP3). For TP1, the scale was different (a five-point Likert scale ranging from 1 = “not at all”, 2 = “barely”, 3 = “moderately”, 4 = “well” to 5 = “very well”) and was therefore mapped to the four-point scale of TP2 and TP3 as follows: 1 = “not at all” was transformed into “no” (0); 2 = “barely” was transformed into “rather no” (1); 3 = “moderately” was transformed into “rather yes” (2); and 4 = “well” and 5 = “very well” were transformed into “yes” (3). In order to compute a percentage of perceived helpfulness, the upper scores of the perceived helpfulness scale were combined (2 = “rather yes” or 3 = “yes”). To operationalize recommendation of the service to others, an adapted version of the net promoter score (NPS (18); was included in the feedback survey. Participants were asked to indicate on a Likert-scale from 0% = “not at all” to 100% = “definitely” how likely they were to recommend *krisenchat* to others.

##### Emotional distress

In the latest sample (TP3), additional items were included in the feedback questionnaire to compare how users felt before and after the chat, using the brief Screening Tool for Psychological Distress (STOP-D (19);. Users retrospectively rated their feelings on five items (depressive symptoms, anxiety, stress, anger, and insufficient social support) on a 10-point Likert scale (0 = “not at all” to 9 = “very much”). A sum score was calculated, resulting in STOP-D pre- and post-values for each participant, with higher scores indicating more emotional distress.

For an overview which measures have been available at which time point, please refer to Table 2.

**Table 2:** Overview of measures included across the three time points.

	Data source	Oct 20**–**Mar 21 (TP1)	Oct 21**–**Mar 22 (TP2)	Oct 22**–**Mar 23 (TP3)
1	Meta Data	Date of chat (time stamp)	Date of chat (time stamp)	Date of chat (time stamp)
2	Information documented by counsellors	Age	Age	Age
		Gender	Gender	Gender
		Current treatment	Current treatment	Current treatment
		Chat Topics	Chat Topics	Chat Topics
3	User feedback	Perceived helpfulness of the service (1 item)	Perceived helpfulness of the service (1 item)	Perceived helpfulness of the service (1 item)
		likelihood of recommending it to others (NPS)	likelihood of recommending it to others (NPS)	likelihood of recommending it to others (NPS)
				STOP-D

Note*.* TP, time point, NPS, net promoter score.

a Topic was deleted in the second and third time period*.* More specific tags were available in the second and in the last time period (e.g., war) but were excluded from the current analyses.

b Topic was newly introduced at timepoint 3 (9th category).

### Statistical analysis

Descriptive statistics were computed for sociodemographic variables (gender, age, current treatment), emotional distress (STOP-D score), user satisfaction (perceived helpfulness and recommendation), and chat topics. Changes in emotional distress before and after the chat session (STOP-D pre and post values, respectively) were examined using Wilcoxon Rank Tests in TP3.

Due to missing data on gender, all analyses on gender (differences) are based on a reduced sample size of overall *N*_TP1_ = 3,404, *N*_TP2_ = 6,327, *N*_TP3_ = 5,132.

Normality was assessed using the Shapiro–Wilk and Kolmogorov–Smirnov tests and visual inspection of normal Q–Q plots. As age was not normally distributed, differences in age between the three time periods were analyzed using the Kruskal–Wallis H test rather than a parametric one-way analysis of variance. User satisfaction (perceived helpfulness and recommendation) was likewise compared across the three time periods using Kruskal–Wallis H tests.

Differences in categorical variables (gender, current treatment, and the eight chat topic categories) between time periods were examined using *χ*^2^ tests. Chat topics by time period and gender were additionally summarized descriptively in tables, and exploratory findings were presented graphically.

All analyses were conducted using IBM SPSS Statistics (Version 29.0). Statistical significance was set at a two-sided *α* = 0.05. When *χ*^2^ tests were significant, pairwise z-tests were performed to identify differences between time periods. Following significant Kruskal–Wallis H tests, pairwise comparisons were conducted using Mann–Whitney U tests with Bonferroni correction for multiple testing. Cramer's V was used to estimate effect sizes for *χ*^2^ tests (small = 0.10, medium = 0.30, large = 0.50) (20), and *r* was reported as the effect size for the non-parametric tests.

## Results

### User characteristics in the latest sample (TP3)

Sociodemographic characteristics and descriptive results of the current sample (TP3) are presented in Table 4. In TP3, the mean age was 17.09 (*SD* = 3.51), with users ranging from 8 to 24 years old. In TP3 (October 2022–March 2023), users ranged from 7 to 24 years of age. The detailed age distribution is presented in Table 4. The largest proportion of users were aged 14–17 years (43.3%), followed by users aged 18–24 years (41.0%). Users aged 7–13 years represented 15.7% of the sample, see also Table 3.

**Table 3:** Age distribution of users in the latest sample (TP3; October 2022–March 2023).

Age group	*n*	%
7–13 years	819	15.7
14–17 years	2,258	43.3
18–24 years	2,141	41.0
Total	5,218	100

**Table 4:** User characteristics across the three time points.

Variable	Oct 20**–**Mar 21 (TP1) *N* = 4,029	Oct 21**–**Mar 22 (TP2) *N* = 6,647	Oct 22**–**Mar 23 (TP3) *N* = 5,218	Test	*p*	Effect size
Age, *M* (SD)	16.04 (3.39)^a^	17.36 (3.46)^b^	17.09 (3.51)^c^	*H(2)* = 374.49	<.001	n.a.
Gender, *n* (%)^x^				*χ*2 (4) = 84.78	<.001	*V* = .076
Female	2,890 (84.9)^a^	5,020 (79.3)^b^	3,953 (77.0)^c^			
Male	453 (13.3)^a^	1,109 (17.5)^b^	988 (19.3)^b^			
Diverse	61 (1.8)^a^	198 (3.1)^b^	191 (3.7)^b^			
Current treatment, *n* (%)	782 (19.4)^a^	1,447 (21.8)^b^	1,227 (23.5)^b^	*χ*2 (2) = 22.52	<.001	*V* = .038
Perceived helpfulness, *M* (SD)	2.47 (0.84)^a^	2.12 (0.81)^b^	2.35 (0.71)^c^	*H*(2) = 276.64	<.001	
Recommendation score, *M* (SD)	8.48 (2.42)^a^	8.09 (2.46)^b^	8.85 (2.00)^a^	*H*(2) = 65.91	<.001	

Note. TP, time point; M, mean; SD, standard deviation; H, Kruskal–Wallis test statistic; *χ*^2^, chi-square statistic; V, Cramér's V; n, number of participants; %, percentage. Different superscript letters (a, b, c) indicate statistically significant pairwise differences within rows (*p* < .05). Percentages marked with ^x^ were calculated using valid cases because of missing data. NPS, net promoter score.

Of the *N* = 5,218 participating chatters in the latest time period (TP3), *n* = 5,133 (98.4%) disclosed their gender. Of those, *n* = 3,953 (77%) identified as female, *n* = 988 (19.3%) as male, and *n* = 191 (3.7%) as diverse. Regarding current treatment, *n* = 1,227 (23.5%) reported already receiving some kind of professional treatment while also receiving counseling from *krisenchat.* In TP3, *N* = 1,473 users participated in the feedback survey and indicated both their user satisfaction and their likelihood of recommending *krisenchat*.

For participants of the current time period (TP3), a Wilcoxon signed rank test showed that the STOP-D mean value was significantly lower after the chat counseling session (*M_post_* = 4.61, *SD_post_* = 2.24) than before the session (*M*_pre_ = 6.48, *SD_pre_* = 1.52), indicating significantly lower emotional distress (*Z* = −27.48, *p* < .001) in chatters.

Perceived helpfulness in TP3 was rated with a mean score of *M* = 2.35, *SD* = 0.71. Overall, *n* = 1,294 (90.1%) of users rated *krisenchat* to have helped them with their concern. The computed recommendation rate for the current sample was 86.5%.

In TP3, the three most frequently reported chat topics were psychosocial distress (54.6%), followed by mental health symptoms (52.9%), and emotional distress (25.7%). The category “war” that was newly introduced in TP3, was reported in *n* = 27 (0.2%).

### User characteristics over time

Compared to the previous time periods, a significant difference in age among *krisenchat* users was found, *H*(2) = 374.49, *p* < .001. Pairwise comparisons revealed that there was a significant age difference between all data periods with the oldest chatters in the second data period (TP2) and the youngest in the first data period (TP1; see also Table 4).

*U*-Tests revealed that age was significantly higher in TP2 compared to TP1, *Z* = −19.17, *p* < .001. Age was significantly higher in TP3 compared to TP2, *Z* = −13.89, *p* < .001. Age was significantly higher in TP1 compared to TP3, *Z* = −4.55, *p* < .001.

As displayed in Table 4, throughout all time periods, the majority of users were female. Compared to the two previous time periods, there were significant differences in gender, *χ*^2^ (4, 14,863) = 84.78, *p* < .001, *V* = 0.053. The *χ*^2^ test showed that the gender distribution significantly differed between all time periods, with the proportion of male and diverse users significantly increasing in TP2 and TP3. The proportion of female users significantly increased from TP1 to TP2, while significantly decreasing in the last period. For males and diverse chatters, there was a significant increase from TP1 to TP2, while no significant differences were found between TP2 and TP3.

A *χ*^2^ test revealed a significant difference between time points in chatters indicating they were currently in treatment, *χ*^2^ (2, 15,894) = 22.52, *p* = <.001, *V* = .038. Chatters in TP3 were significantly more likely to be in current treatment compared to the previous two data periods, while chatters in TP1 and TP2 did not differ concerning current treatment.

Compared to the previous time periods, a significant difference in recommendation rates was found, *H*(2) = 65.91, *p* < .001. Pairwise comparisons revealed that recommendation rate was significantly higher in TP2 compared to TP1, *Z* = −6.93, *p* < .001. Recommendation rate was significantly higher in TP3 compared to TP1, *Z* = −0.39, *p* < .001. Recommendation rate was also significantly higher in TP3 compared to TP2, *Z* = −6.63, *p* < .001.

The results of the comparison of perceived helpfulness across the three time periods are presented in Table 4. A significant difference in perceived helpfulness scores was observed between the three time periods, *H*(2) = 276.64, *p* < .001. *post hoc* pairwise comparisons revealed significant differences between all three time periods. Compared with TP1, sessions in TP2 were rated as significantly less helpful (*Z* = −16.17, *p* < .001). Similarly, perceived helpfulness ratings were significantly lower in TP3 compared with TP1 (*Z* = −7.75, *p* < .001). In contrast, ratings in TP3 were significantly higher compared with TP2 (*Z* = −8.66, *p* < .001). Overall, perceived helpfulness decreased from TP1 to TP2, followed by a partial increase in TP3, although ratings remained below the level observed in TP1, please see Table 5.

**Table 5:** Changes in categories over time. Summary of overall chi-square tests and pairwise comparisons.

Category	Overall test	Pairwise comparisons	Direction of change	Effect size (V)
Mental Health Symptoms	*χ*^2^ (2, 15,894) = 47.73, *p* < .001	TP2 vs. TP3	Decrease TP2 → TP3	.055
Emotional Distress	*χ*^2^ (2, 15,894) = 33.14, *p* < .001	TP2 vs. TP3	Increase TP2 → TP3	.046
Psychosocial Distress	*χ*^2^ (2, 15,894) = 567.39, *p* < .001	TP1 vs. TP2; TP2 vs. TP3	Increase TP1 → TP2, decrease TP2 → TP3	.189

Note. TP, time point; *χ*^2^, chi-square test; V, Cramér's V. Pairwise comparisons refer to *post hoc* comparisons. Non-significant comparisons are omitted.

### Changes in chat topics over time

Compared to the previous time periods, *χ*^2^ tests revealed a significant decrease in the chat topic “Mental Health Symptoms” between TP2 and TP3, *χ*^2^ (2, 15,894) = 47.73, *p* < .001, *V* = .055, while no significant differences between the first two periods were found.

Concerning “Emotional Distress”, there was significant association between topic and time period, *χ*^2^ (2, 15,894) = 33.14, *p* = <.001, *V* = .046. Pairwise comparison revealed a significant increase from TP2 to TP3, while no differences were found between TP1 and TP2.

For “Psychosocial Distress”, significant differences were found, *χ*^2^ (2, 15,894) = 567.39, *p* = < .001, *V* = .189. Pairwise comparisons revealed a significantly increasing proportion of this topic from TP1 to TP2 followed by a significant decrease from TP2 to TP3. For “Suicidality”, significant differences were found, *χ*^2^ (2, 15,894) = 21.375, *p* = < .001, *V* = .037. Pairwise comparisons revealed no significant differences between TP1 and TP2, while there was a significant decrease from TP2 to TP3.

**Figure 1:**
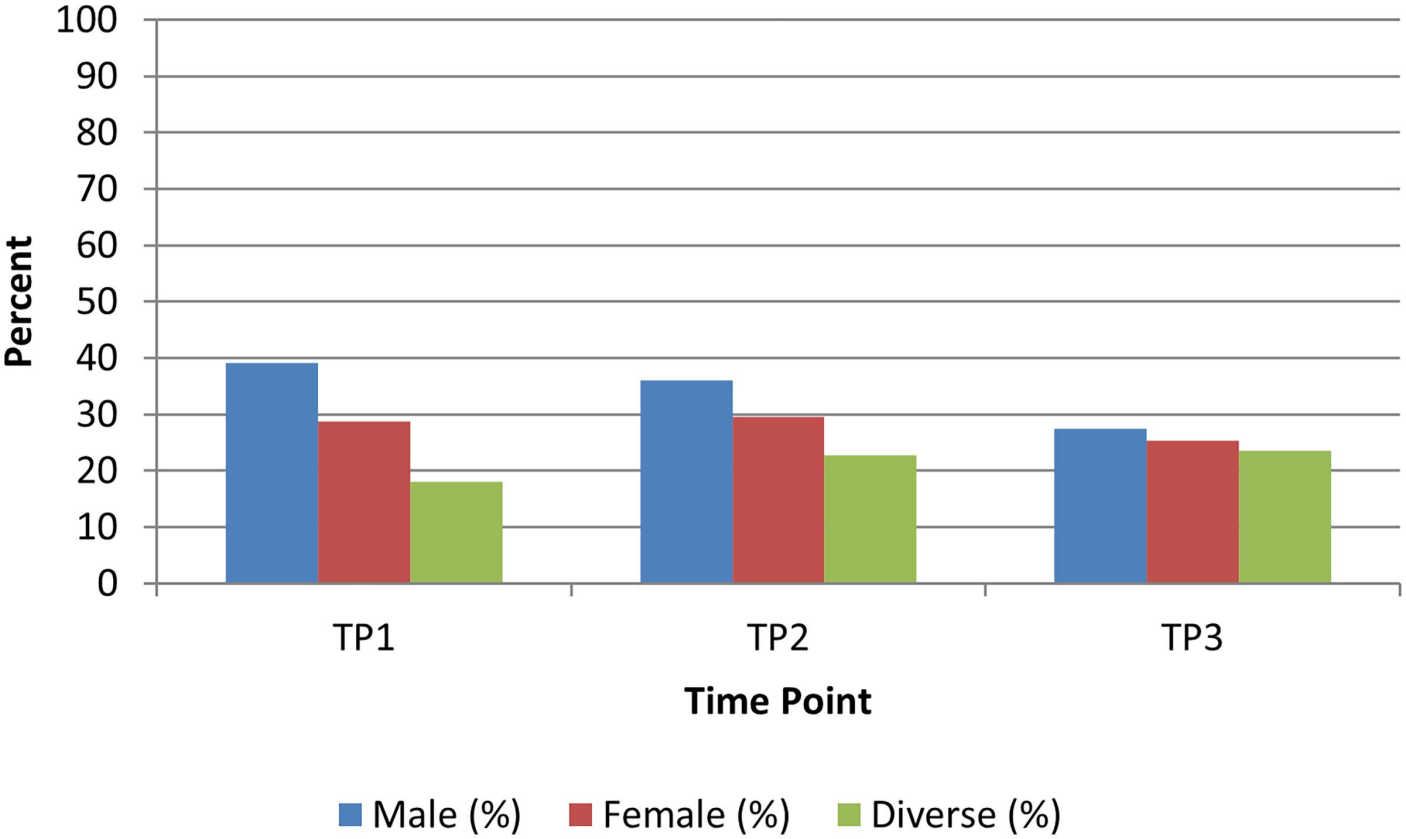
Chat category “Emotional Distress” over time and with respect to all gender identities.

**Figure 2:**
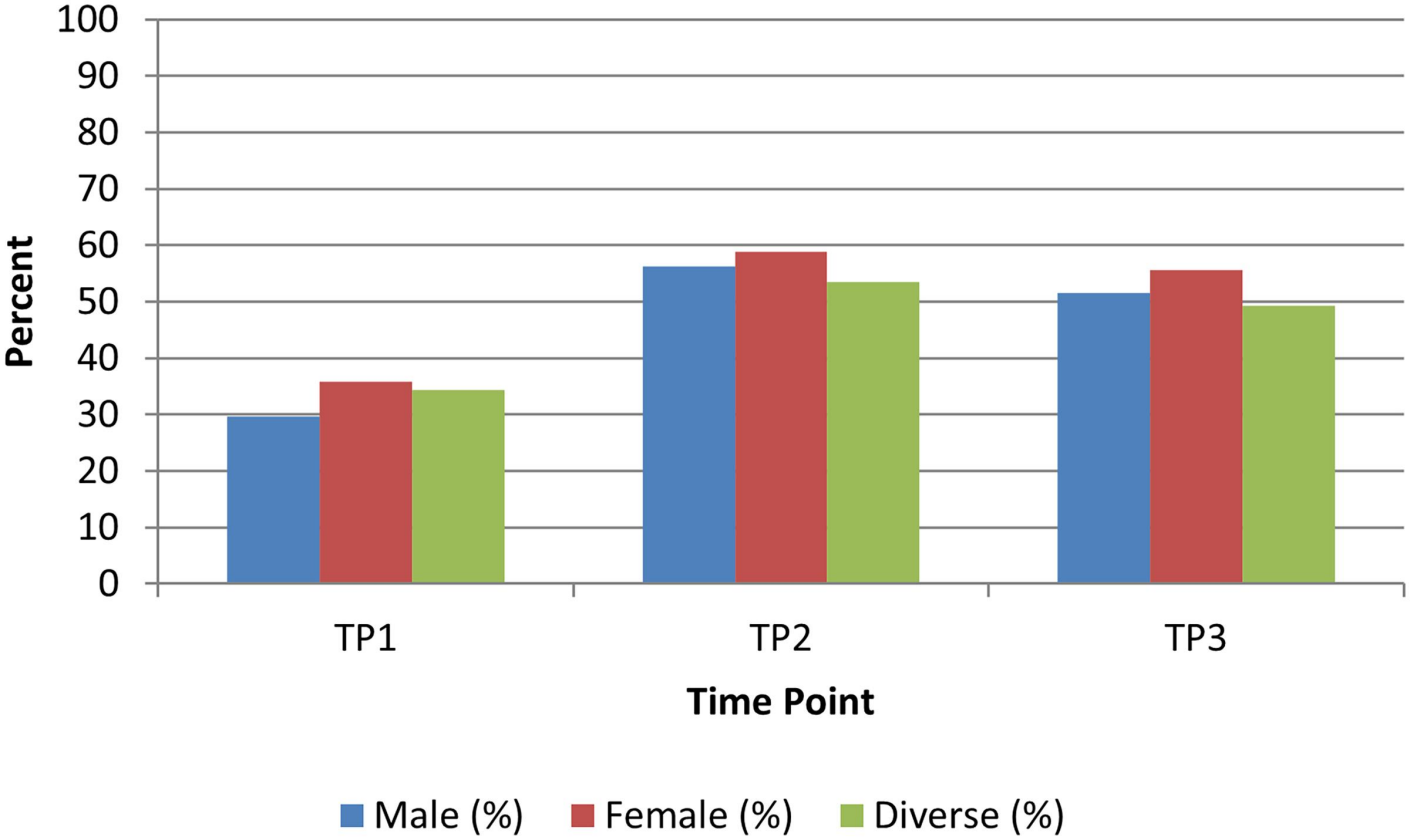
Chat category “Psychosocial Distress” over time and with respect to all gender identities.

**Figure 3:**
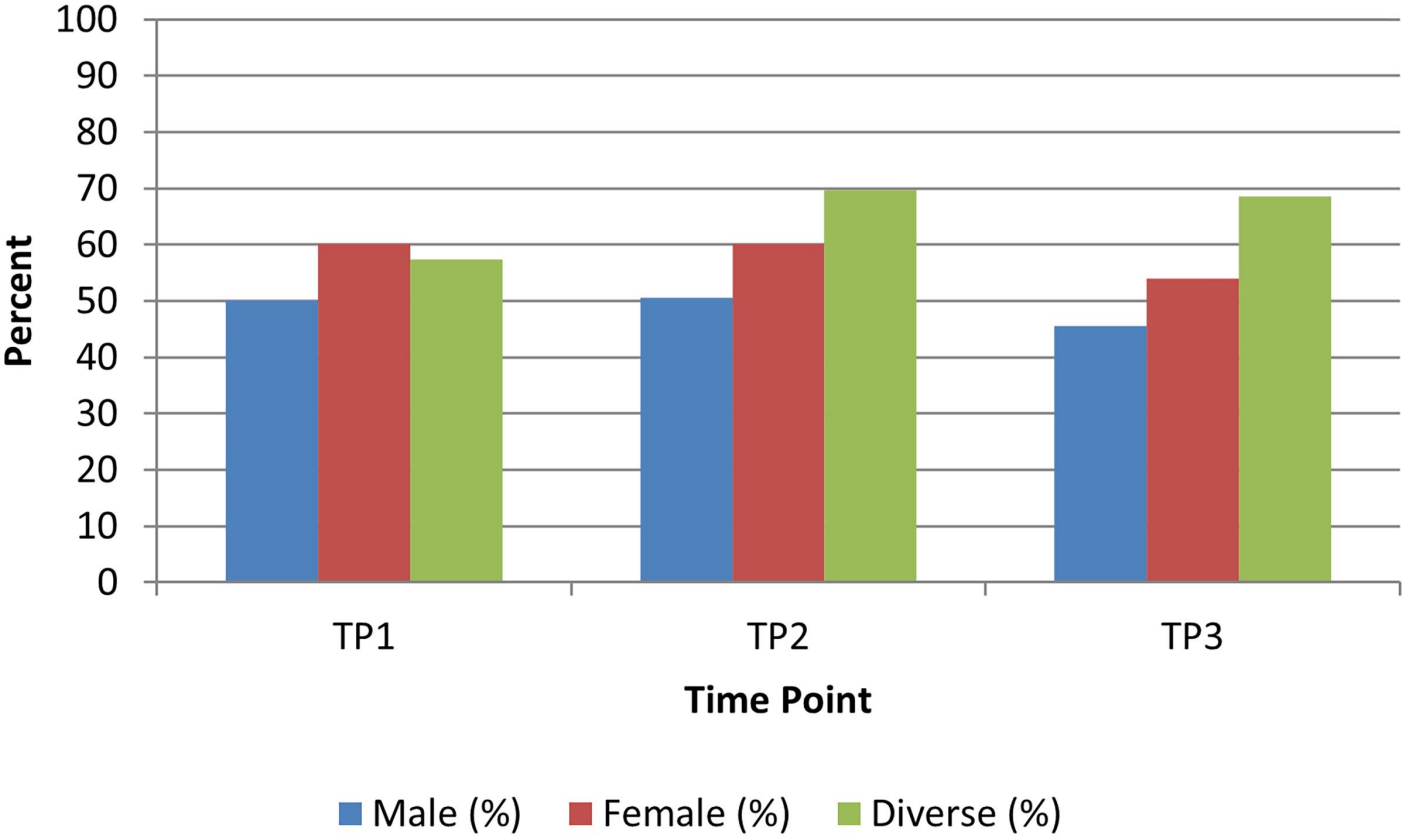
Chat category “Mental Health Symptoms” over time and with respect to all gender identities.

The top three chat categories and crisis related themes are displayed in Table 6. “Emotional distress” was consistently a prominent topic across all time periods and genders, though its prevalence slightly decreased over time, particularly among males (from 39.1% in TP1 to 27.4% in TP3). “Psychosocial distress” increased between TP1 and TP2 across all genders, with the highest rates in females (from 35.8% in TP1 to 58.8% in TP2). However, there was a slight decline in TP3. Females consistently reported the highest levels of “Emotional distress”, “Psychosocial distress”, and “Mental Health Symptoms”. Males showed a decrease in “Emotional Distress” and “Mental Health Symptoms” over time, while reporting relatively stable rates of “Psychosocial Distress”. For a graphic display of the top three chat categories with respect to time point and gender identity, please consult Figures 1–3.

**Table 6:** Top three chat categoriess and crisis-related themes across the three time points.

Variable	Oct 20**–**Mar 21 (TP1) *N* = 4,029	Oct 21**–**Mar 22 (TP2) *N* = 6,327	Oct 22**–**Mar 23 (TP3) *N* = 5,218
Categories/Rank (%)	1 Mental Health Symptoms (58.6)	1 Mental Health Symptoms (58.7)	1 Psychosocial Distress (54.6)
	2 Psychosocial Distress (35.0)	2 Psychosocial Distress (57.9)	2 Mental Health Symptoms (52.9)
	3 Emotional Distress (29.5)	3 Emotional Distress (30.4)	3 Emotional Distress (25.7)
Crisis related themes/Rank (%)	1 Suicidality (19.7)	1 Suicidality (19.1)	1 Suicidality (16.4)
	2 Corona (10.5)	2 Loneliness (7.7)	2 Loneliness (5.4)
	3 Loneliness (8.5)	3 Corona (2.4)	3 Corona (0.2)

Values are presented as *n* (%). Percentages refer to participants within each gender group at each time point. TP, time point.

“Loneliness” and “Lovesickness” showed a downward trend over time for all genders. “Mental Health Symptoms” remained a dominant topic, especially among females and diverse individuals, though there was a slight decline among males in TP3. The topics “Depression” and “Suicidality” rates remained relatively stable, though suicidality was consistently higher among diverse individuals, peaking at 28.2% in TP2.

Overall, gender diverse individuals reported the highest rates of mental health concerns across all three time periods. “LGBTQIA+” issues were most commonly reported in diverse gender dimension, though mentions decreased over time (from 60.7% in TP1 to 18.8% in TP3). The “Covid-19” topic declined sharply across all genders, dropping to nearly zero by TP3.

Individuals of diverse gender identity most frequently received the tags “suicidality” and “LGBTQIA+” concerns, though both declined over time.

The observed changes in chat topics over the three time periods occurred alongside major changes in the broader social context. While COVID-19-related concerns became less frequent over time, psychosocial distress and relationship-related topics gained prominence, particularly in TP3. This shift may reflect changing challenges faced by young people during the later stages of the pandemic, including prolonged social disruption, reduced interpersonal contact, and associated emotional strain. Although these findings cannot establish causal relationships, they suggest that patterns of digital mental health service use may change in response to evolving external stressors. With regard to time point and gender, all chat categories and topics of interest are descriptively displayed in Table 7.

**Table 7:** Distribution of chat categories and crisis-related topics by gender identity across the three time points.

Categories/Topics	Oct 20**–**Mar 21 (TP1)		Diverse *n* = 61	Oct 21**–**Mar 22 (TP2)		Diverse *n* = 198	Oct 22**–**Mar 23 (TP3)		Diverse *n* = 191
	Male *n* = 453	Female *n* = 2,890		Male *n* = 1,109	Female *n* = 5,020		Male *n* = 988	Female *n* = 3,953	
**Emotional distress**	**177 (39.1)**	**833 (28.8)**	**11 (18.0)**	**400 (36.1)**	**1,487 (29.6)**	**45 (22.7)**	**271 (27.4)**	**1,006 (25.4)**	**45 (23.6)**
Loneliness	59 (13.0)	235 (8.1)	3 (4.9)	130 (11.7)	342 (6.8)	11 (5.6)	62 (6.3)	210 (5.3)	8 (4.2)
Lovesick	93 (20.5)	448 (15.5)	4 (6.6)	163 (14.7)	462 (9.2)	9 (4.5)	115 (11.6)	341 (8.6)	13 (6.8)
Self-worth	10 (2.2)	71 (2.5)	0	100 (9.0)	493 (9.8)	21 (10.6)	58 (5.9)	327 (8.3)	9 (4.7)
**Psychosocial distress**	**134 (29.6)**	**1,036 (35.8)**	**21 (34.4)**	**624 (56.3)**	**2,950 (58.8)**	**106 (53.5)**	**509 (51.5)**	**2,197 (55.6)**	**94 (49.2)**
Mobbing	**19 (4.2)**	**139 (4.8)**	**7 (11.5)**	**56 (5.0)**	**216 (4.3)**	**19 (9.6)**	**46 (4.7)**	**226 (5.7)**	**15 (7.9)**
**Mental health symptoms**	**227 (50.1)**	**1,738 (60.1)**	**35 (57.4)**	**560 (50.5)**	**3,015 (60.1)**	**138 (69.7)**	**450 (45.5)**	**2,131 (53.9)**	**131 (68.6)**
Depression	89 (19.6)	602 (20.8)	8 (13.1)	230 (20.7)	1,125 (22.4)	36 (18.2)	154 (15.6)	615 (15.6)	36 (18.8)
Suicidality	83 (18.3)	548 (20.2)	16 (26.2)	187 (16.9)	977 (19.5)	56 (28.2)	152 (15.4)	644 (16.3)	44 (23.0)
**LGBTQA+**	**29 (6.4)**	**70 (2.4)**	**37 (60.7)**	**82 (7.4)**	**85 (1.7)**	**65 (32.8)**	**59 (6.0)**	**47 (1.2)**	**36 (18.8)**
**Covid-19**	**60 (13.2)**	**314 (10.9)**	**4 (6.6)**	**27 (2.4)**	**123 (2.5)**	**3 (1.5)**	**4 (0.4)**	**5 (0.1)**	**0 (0)**
**Education**	**11 (2.4)**	**71 (2.5)**	**1 (1.6)**	**87 (7.8)**	**380 (7.6)**	**18 (9.8)**	**19 (9.1)**	**70 (1.8)**	**5 (2.6)**
**Sexual harassment**	**12 (2.6)**	**180 (6.2)**	**3 (4.9)**	**16 (1.4)**	**267 (5.3)**	**9 (4.5)**	**19 (1.9)**	**202 (5.1)**	**4 (2.1)**
**Violence**	**17 (3.8)**	**261 (9.0)**	**4 (6.6)**	**57 (5.1)**	**321 (6.4)**	**11 (5.6)**	**57 (5.8)**	**460 (11.6)**	**12 (6.3)**
**War**							**12 (1.2)**	**14 (0.4)**	**0 (0)**

Bold text corresponds to categories.

## Discussion

The results of this study demonstrate a high feasibility of *krisenchat* over three consecutive timepoints: the service remained highly acceptable, feasible, and the majority of users remained highly satisfied throughout the pandemic and in post-pandemic periods, from 2020 until 2023. This study is one of the first to consider female, male and diverse gender dimensions, integrate them into gender analyses, and further explore data on chat topics within an online chat counselling context. To our knowledge, this is also the first systematic observation of chat topics in a counseling service over time and in relation to different gender identities.

The overall findings provide insights into changes over time and the general robustness of *krisenchat* in the context of collective life events. This further adds to the evidence base of prior findings on the service, all of which were conducted using cross-sectional samples at specific time points (13–15, 21, 22).

### Effects on emotional distress in the most recent sample (TP3)

A key finding that adds to previous research is the significantly positive effect of chat sessions on emotional distress in the most recent, post-pandemic sample (TP3). This result favors the service, indicating a positive short-term effect of the sessions. Additionally, this finding supports the usability and reliability of the STOP-D instrument, which has not yet been widely used for research purposes in mental health but rather as a brief psychosocial screening tool initially employed in outpatient cardiology settings (19). Telephone line, chat-based interactions as well as newly developed chatbot delivered interventions can have positive effects on emotional distress and related mental health outcomes (23, 24).

### User characteristics over time

In all time periods, *krisenchat* was primarily accessed by young females, though the service experienced a significant increase in users identifying as male and diverse from TP1 to TP2. A possible reason for this shift could be intensified public relations work by *krisenchat* towards these gender identities from 2021 on. Additionally, these known as more vulnerable, yet less help-seeking individuals might have benefitted more from the anonymous support offered by *krisenchat*. This trend concerning young males could also reflect a broader societal shift, where male users and those of diverse gender identities sought help more than they would have without the presence of an online service (25). *Krisenchat* as a low-threshold and anonymous service may be of particular importance to those identifying themselves as diverse. The polycrisis has been linked to adverse mental health outcomes among college students, particularly those from disadvantaged groups (e.g., by gender, sexual orientation), who experience worse mental health, higher negative affectivity, depressive symptoms, and poorer subjective physical and mental health compared to their peers (11).

In TP2, *krisenchat* users were significantly older—by more than a year on average—compared to TP1. However, after the pandemic (from TP2 to TP3), the average age significantly decreased. Despite this decline, users in TP3 were still older than those in the first year of the service (TP1). Overall, the average user across all time points is 17 years old and female, consistent with cross-sectional findings of previous studies on the service (13).

A potential factor contributing to this shift is the change in *krisenchats* marketing strategies. While in TP2, marketing was primarily focusing on Instagram, a platform predominantly used by older adolescents, TP3 faced a transition to TikTok, which is more popular among younger audiences (26). Alternatively, the aging of the user base could be explained by the continued engagement of young individuals who initially sought support from *krisenchat* and continued to use the service over the years. Despite some similarities, differences in usage patterns emerged across time periods: The significant increase in users reporting being currently in treatment over all three points in time suggests a higher need for online counseling as an additional source of support. As the effects of the pandemic persisted, this increase also points to a potential higher overall burden in the post-pandemic sample (Oct 2022**–**Mar 2023). The results indicate that the service meets the needs and expectations of the target group, as evidenced by the high user satisfaction rates over time and the increasing recommendation rate of over 80% across all three time periods.

### Recommendation and perceived helpfulness over time

Perceived helpfulness first significantly decreased from TP1 to TP2, then significantly increased from TP2 to TP3 while remaining high, in general. The highest overall perceived helpfulness in TP3 could be attributed to several factors, including increased need for support, higher pressure on the health care system at the time, and potential improvements in training concepts for counsellors (27), which may have led to a general increase in the counselling quality of the service.

### Chat topics over time

The analysis of chat contents over time revealed a broad range of concerns addressed by users of *krisenchat*. Across the whole observation period, “Mental Health Symptoms” was the most frequently identified topic, with more than half of chats receiving this tag from a counsellor during the first two time periods. However, changes emerged when comparing the individual time periods. While “Mental Health Symptoms” remained the predominant concern in TP1 and TP2, a shift was observed in TP3, when “Psychosocial Distress” became the most frequently reported topic, followed by “Mental Health Symptoms”. Thus, although the relative prominence of topics changed over time, mental health-related concerns remained a central theme throughout the pandemic.

The variety of topics identified is consistent with previous findings from youth helplines, where calls are commonly driven by a broad range of psychosocial concerns rather than a single issue (17). In TP3, interactional and relationship-related topics were reported more frequently than topics directly related to users' mental health. This shift may reflect the longer-term consequences of almost two years of pandemic-related isolation and reduced social interaction. It is in line with previous research showing that 24% of individuals reported a deterioration in relationship quality, as well as research highlighting the widespread prevalence of loneliness and its predominantly negative effects on mental health (28, 29).

Regarding specific crisis-related themes, “Loneliness” was reported less frequently over time, while “Corona”-related concerns declined markedly from 10% to less than 1%. Suicidality remained prevalent during the first two periods but decreased in TP3, consistent with findings on suicidal ideation and suicide attempts following the COVID-19 outbreak (30).

### Practical implications

The observed fluctuations in age and gender composition over time suggest that digital mental health services such as *krisenchat* should adopt flexible outreach and design strategies that can respond to changing user populations. The shift toward younger users in TP3 highlights the importance of platform-specific outreach and youth-oriented, mobile-first interface design.

The increasing proportion of male and gender-diverse users underscores the need for explicitly inclusive and gender-sensitive counselling approaches, including the consistent use of inclusive language, non-binary gender options, and counsellor training that addresses minority stress, identity-related concerns, and differing help-seeking patterns. Changes in reported chat topics and perceived helpfulness over time indicate that counsellor training should be continuously adapted to evolving user needs. Increased focus on psychosocial distress, relationship issues, and post-pandemic stressors suggests a need for enhanced competencies in addressing interpersonal difficulties and more chronic emotional strain. The improvement in perceived helpfulness in TP3 further highlights the potential impact of updated training concepts, supporting ongoing investment in training, supervision, and quality assurance to maintain service effectiveness during periods of collective stress.

### Strengths and limitations

To the best of our knowledge, this study is, to date, the only effort to utilize supply data in assessing the utilization behavior and conducting exploratory research on chat topics over time for a messenger-based chat counseling service among German-speaking youths and young adults. However, several limitations need to be addressed: We employed a retrospective study design and relied on convenience sampling. Most of the instruments used are not standardized measurement tools, and this study did not include follow-up data. The tags over time (as displayed in Table 1) underwent changes that might have affected the informative value (i.e., validity) of the overall categories. Further, the use of a non-random sample introduces potential sampling bias, which may limit the generalizability of the findings, particularly to non-German-speaking populations. Interpretation of the results is further constrained by tagging inconsistencies, as chat topics were identified through counselor-generated tags, introducing subjectivity and potential variability. Additionally, the retrospective self-assessment of distress using the STOP-D may be subject to a response bias, including recall bias and social desirability effects, which may have affected the accuracy of reported distress levels. The choice of a retrospective rather than real-time assessment should therefore be interpreted cautiously. Moreover, the STOP-D was originally developed and validated in cardiology settings, and its application in a psychosocial context remains insufficiently validated. Future research should prioritize prospective assessment designs, standardized tagging procedures, and further validation of measurement tools to reduce bias and strengthen the robustness of findings.

## Conclusion

Our study examined shifts in user activity and engagement within a digital mental healthcare service throughout the COVID-19 pandemic and beyond. The results indicate that young people's usage patterns in digital mental health services may be influenced by external stressors, such as a pandemic or the recent polycrisis in terms of reasons why young people reach out. These patterns should be monitored further to inform counselor training, design, and possibly personalize online counseling interventions. In conclusion, future research should focus on longitudinal studies to assess the long-term effects of interventions, as well as the validation of tools like the STOP-D in youth populations. The integration of AI-driven support systems could enhance personalized care and improve service delivery efficiency.

## Data Availability

The raw data supporting the conclusions of this article will be made available by the authors, without undue reservation.
